# Temporal Expression of Bacterial Proteins Instructs Host CD4 T Cell Expansion and Th17 Development

**DOI:** 10.1371/journal.ppat.1002499

**Published:** 2012-01-19

**Authors:** Seung-Joo Lee, James B. McLachlan, Jonathan R. Kurtz, Danhua Fan, Sebastian E. Winter, Andreas J. Baumler, Marc K. Jenkins, Stephen J. McSorley

**Affiliations:** 1 Center for Comparative Medicine, School of Veterinary Medicine, University of California Davis, Davis, California, United States of America; 2 Center for Immunology, University of Minnesota Medical School, Minneapolis, Minnesota, United States of America; 3 Department of Microbiology & Immunology, Tulane University Health Science Center, New Orleans, Louisiana, United States of America; 4 Department of Biostatistics and Informatics, University of Minnesota, Minneapolis, Minnesota, United States of America; 5 Department of Medical Microbiology and Immunology, School of Medicine, University of California Davis, Davis, California, United States of America; University of Pennsylvania, United States of America

## Abstract

Pathogens can substantially alter gene expression within an infected host depending on metabolic or virulence requirements in different tissues, however, the effect of these alterations on host immunity are unclear. Here we visualized multiple CD4 T cell responses to temporally expressed proteins in *Salmonella*-infected mice. Flagellin-specific CD4 T cells expanded and contracted early, differentiated into Th1 and Th17 lineages, and were enriched in mucosal tissues after oral infection. In contrast, CD4 T cells responding to *Salmonella* Type-III Secretion System (TTSS) effectors steadily accumulated until bacterial clearance was achieved, primarily differentiated into Th1 cells, and were predominantly detected in systemic tissues. Thus, pathogen regulation of antigen expression plays a major role in orchestrating the expansion, differentiation, and location of antigen-specific CD4 T cells in vivo.

## Author Summary

Pathogens alter protein expression in an infected host, depending on metabolic or virulence requirements, but the effect of these changes on the immune response is unclear. We identified new class-II epitopes within *Salmonella* type-III secretion system effector proteins and generated a methodology to visualize endogenous T cells responding to these epitopes. Our study shows that *Salmonella* flagellin generates a mixed Th1 and Th17 response that contracts early and is enriched in mucosal tissues. In contrast, we found that *Salmonella* T3SS effectors generate a sustained Th1 response that requires a persisting infection and is enriched in systemic tissues. These data demonstrate that in vivo antigen regulation substantially alters the antigen specificity, helper differentiation, and anatomical location of pathogen-specific CD4 T cells.

## Introduction

Generating vaccines for current and emerging infectious diseases remains an important goal of immunological research [1], [2]. An ideal vaccine will induce the expansion and maturation of naïve pathogen-specific lymphocyte clones that exist at low frequency in uninfected or unimmunized individuals [3]. During infection or immunization, pathogen-specific T cells expand within secondary lymphoid tissues after recognition of foreign peptides in the context of host MHC molecules. These dividing pathogen-specific T cells acquire effector capabilities that are tailored to combat different classes of microbial pathogen [4], [5]. Following the resolution of primary infection, a cohort of these expanded pathogen-specific T cells persists to provide robust secondary immunity against another encounter with the same pathogen [6].

Naïve CD4 T cells can differentiate into T helper 1 (Th1), Th2, or Th17 effector lineages depending on the instructional cues delivered during initial activation [7]. Th1 cells express the transcription factor T-bet, secrete IFN-γ, and protect the host against infection with intra-macrophage pathogens [8], [9]. In contrast, Th2 cells express GATA-3, secrete IL-4, and combat large extracellular pathogens [10], [11]. Th17 cells are a recently described effector cell lineage that express RORγt, secrete IL-17, and contribute to clearance of extracellular bacterial and fungal infections [5], [12]. A number of variables can influence CD4 T helper cell differentiation, including TCR affinity for peptide/MHC, antigen dose, costimulatory signals, and the local concentration of inflammatory cytokines [7].

In vivo analysis of CD4 T cell differentiation during infection has often involved visualization of adoptively transferred TCR transgenic T cells [13]–[19]. Although this experimental approach allows detection of pathogen-specific T cells using conventional methodologies, it can also introduce experimental variables that are not present in a natural host [20]–[22]. The development of peptide-MHC tetramer enrichment methodologies now allows direct visualization of low frequency antigen-specific CD4 T cell populations without requiring adoptive transfer of TCR transgenic cells [23]. Using this approach, infection via the intra-nasal route was shown to enhance development of *Listeria*-specific Th17 cells while systemic infection encouraged Th1 development [24]. Most pathogens differentially regulate their protein expression in mucosal or systemic tissues depending on local metabolic or virulence requirements [25], [26], but it is not yet clear how this might affect CD4 T cell expansion and differentiation to stage-specific antigens expressed in different tissues.

Here, we examined this issue using peptide-MHC tetramers and ELISPOTs that allow simultaneous tracking of CD4 T cell responses to *Salmonella* flagellin and type-III-secretion system (TTSS) effector proteins. We found that flagellin-specific CD4 T cells undergo early expansion and contraction and after oral infection generate Th17 cells that localize to infected mucosal tissues. In contrast, CD4 T cells responding to TTSS effector proteins expanded and accumulated over several weeks and primarily differentiated to a Th1 lineage localized in systemic tissues. Thus, an infected host simultaneously develops distinct populations of CD4 effector lineages that detect stage-specific antigens and have divergent anatomical localization.

## Results

### Identification of novel class-II *Salmonella* epitopes

**Figure 1:**
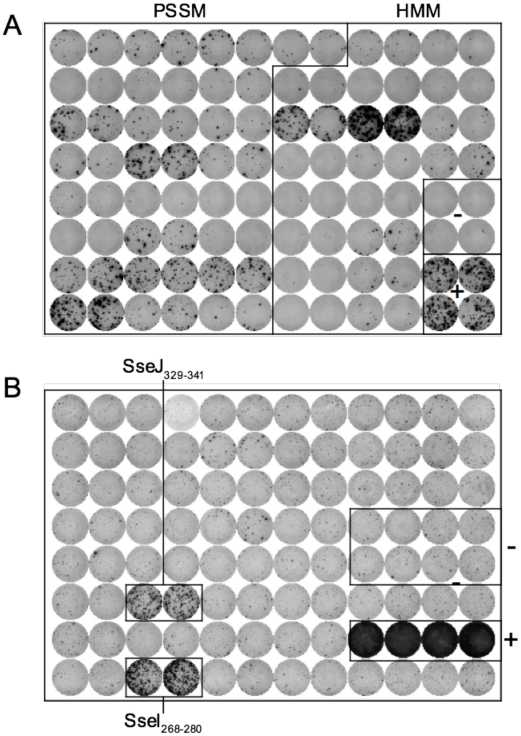
Discovery of novel *Salmonella* I-A^b^ epitopes. (A) C57BL/6 mice were immunized with a mixture of 4 groups of 11 peptides from Table 1 at 5 µg/peptide in CFA. Eight days later, draining lymph nodes were isolated and purified CD4 T cells restimulated with individual peptides (Table 1) in the presence of irradiated splenocytes. IFN-γ production was measured 24–36 hours later by ELISPOT. (B) 129Sv mice were orally infected with virulent 5x10^7^ *Salmonella* (SL1344) and spleens harvested 11 weeks later. Purified CD4 T cells were restimulated with peptides from Table 2 for 24–36 h in the presence of irradiated splenocytes and IFN-γ production measured by ELISPOT. ConA and no peptide wells constitute the positive and negative controls respectively. Each plate is representative of 2–3 individual experiments.

I-A^b^ epitopes have been identified within *Salmonella* flagellin [27], [28], and these remain the only confirmed class-II epitopes for examining CD4 response to *Salmonella* in C57BL/6 mice [29]. However, flagellin is an unusual antigen that can be directly detected by the innate immune system, and is also rapidly down-regulated as bacteria transition to intracellular growth [30]–[32]. In order to uncover antigens that are recognized by CD4 T cells during intracellular bacterial growth, we used a bioinformatic approach to identify novel *Salmonella* I-A^b^ epitopes. Based on the published structure of I-A^b^ in complex with human class-II invariant chain peptide (CLIP) [33], we used a positional-specific scoring matrix (PSSM) and Hidden Markov Model (HMM) to interrogate all open reading frames in the *Salmonella* genome for I-A^b^ epitopes. Although this yielded numerous epitopes that were then confirmed by peptide immunization (Table 1 and Figure 1A), none were found to be natural epitopes during murine *Salmonella* infection or after immunization with heat-killed *Salmonella* (data not shown). Since many of these candidate peptides were derived from cytoplasmic proteins that are unlikely to enter the class-II presentation pathway, we modified our approach to focus on outer membrane or secreted *Salmonella* proteins, (Table 2), many of which are required for bacterial persistence in vivo [34]. Using this strategy we identified natural I-A^b^ epitopes in two effector proteins that are encoded by *Salmonella* Pathogenicity Island 2 (SPI2) TTSS, SseI and SseJ (Figure 1B and Table 3).

**Table 1:** Salmonella peptides screened for immunogenicity in C57BL/6 mice.

	Protein ID	Start Site	a.a sequence	Protein source	
Group 1	AAL21503.1	385	ihyaapvpervvy	Gifsy-1 prophage protein	Position-specific scoring matrix (PSSM)
	AAL21353.1	399	arypnphpllvva	CPPZ-55 prophage protein	
	AAL19315.1	232	lyyflpvpvlvla	cytochrome BD2 subunit II	
	AAL19990.1	525	nvyfqpvpallce	aminopeptidase N	
	AAL22132.1	186	saylgpvavlvdt	PTS family galactitol-specific enzyme IIC	
	AAL19496.1	255	asyitpvpggvgp	5,10-methylene-tetrahydrofolate dehydrogenase	
	AAL20821.1	105	daykaptpvevve	putative SAM-dependent methyltransferase	
	AAL21007.1	177	gvyhdpkpggvsa	glucose-1-phosphate transferase	
	AAL23362.1	74	gfyeepeaapvai	primosomal protein I	
	AAL19397.1	636	vdyyvpvpeveki	cytochrome o ubiquinol oxidase subunit I	
	AAL22828.1	128	igyigpvperalq	proline dipeptidase	
Group 2	AAL22133.1	290	mnysapwpgeewe	galactitol-1-phosphate dehydrogenase	
	AAL23344.1	125	kdydfpvpmfvlp	endonuclease R	
	AAL20285.1	298	aiylypsplicna	putative MFS family transport protein	
	AAL20249.1	92	mdylsprpldahn	putative cytoplasmic protein	
	AAL19903.1	50	alyfipyalivgq	putative APC family, amino-acid transporter	
	AAL19268.1	56	psyhspiaklvqn	putative glutamine amidotransferase	
	AAL20131.1	208	spylapvphrgke	putative metal-dependent hydrolase	
	AAL23330.1	137	gayhvpsptitgs	isoaspartyl dipeptidase	
	AAL23343.1	149	gqyftprplikti	DNA methylase M, host modification	
	AAL21058.1	620	saypdpqaligrq	methionine tRNA synthetase	
	AAL20683.1	272	tgyhnpvalklgl	putative invasin	
Group 3	AAL20248.1	73	fwynsptpnfqgs	putative regulator	
	AAL19422.1	499	wlyskplpatafi	putative diguanylate cyclase/phosphodiesterase domain 2	
	AAL19504.1	23	cryhsptpqifdr	putative regulatory protein	
	AAL19892.1	475	qgvdtplgnhasr	cytochrome-related transporter	Hidden Markov model (HMM)
	AAL23273.1	511	qewhpphikgqwq	trehalose-6-P hydrolase	
	AAL22827.1	697	plyevpeglrnka	3-hydroxyacyl-coA dehydrogenase of 4-enzyme FadB protein	
	AAL22180.1	118	vgfedpelgtsil	pyruvate ABC superfamily transport protein	
	AAL22114.1	247	ygydisrparnaq	pyruvate formate-lyase 4	
	AAL19485.1	16	adyrqpwqmsgvw	putative cytoplasmic protein	
	AAL23296.1	398	msnhnplahelig	aminopeptidase A	
	AAL19180.1	213	slylgavaatvre	30S ribosomal subunit protein S2	
Group 4	AAL20696.1	55	glytsavagivia	putative SulP family transport protein	
	AAL19899.1	653	eiysqaladiaat	anaerobic dimethyl sulfoxide reductase, subunit A	
	AAL19178.1	370	lfirepqailrmf	uridylyltransferase	
	AAL22968.1	304	grydtsaldemk	outer membrane receptor for transport of vitamin B12	
	AAL21333.1	130	tlfhvpvtemacq	putative membrane carboxypeptidase	
	AAL20667.1	559	vmyehpethfeel	iron-dependent alcohol dehydrogenase sn-glycerol-3-phosphate dehydrogenase (anerobic)	
	AAL21186.1	349	qhffdpqpwqqfg	membrane anchor subunit	
	AAL22520.1	163	evfswaatqvvta	D-xylose isomerase	
	AAL21756.1	110	pvvvvsppapqpt	invasion genes transcription activator	
	AAL19161.1	20	ggygsaqaaiapd	putative periplasmic fimbrial chaperone	
	AAL22804.1	36	hgyrgavasiagl	homoserine/homoserine lactone efflux protein	

**Table 2:** List of outer membrane and secreted *Salmonella* class-II epitopes tested.

Protein_ID	Start_site	a.a. sequence	Protein source
AAL20026.1	71	nnfswplpetamr	PipD
AAL20026.1	355	iaygmpslsvylp	PipD
AAL22477.1	183	psylppqlaqyfs	endo-1,4-D-glucanase
AAL22477.1	195	srfgapwstlret	endo-1,4-D-glucanase
AAL19497.1	22	avaadptpvsvsg	FimA
AAL19497.1	162	arykataaattpg	FimA
AAL20501.1	166	aynalpqalgaip	SteA
AAL20501.1	180	leyyiarasnmqe	SteA
AAL19906.1	90	keyanpsqqdrfv	SopD2
AAL19906.1	257	qaykpslssdlie	SopD2
AAL20777.1	219	tlylgafagvfgi	PagO
AAL20777.1	201	wfferpqvstfsv	PagO
AAL20549.1	9	qgyftssissekf	SseJ
AAL20549.1	331	cyyetadafkvim	SseJ
AAL19985.1	245	snlngplilsade	SseI
AAL19985.1	270	liyytdfsnssia	SseI
AAL20174.1	52	yryeddspvsfis	PagC
AAL20174.1	164	vgyegsnisstki	PagC
AAL21040.1	35	ptlsppssghvsf	SseK2
AAL21040.1	88	npylnniinaaii	SseK2
AAL20322.1	43	egygvllillmvi	SseB
AAL20322.1	35	lsqqnpfaegygv	SseB
AAL21825.1	193	rpaeapdhplvew	SopD
AAL21825.1	13	qnytlnesrlahl	SopD
AAL21169.1	345	styvdykinlldk	OmpC
AAL21169.1	231	rlygngdratvyt	OmpC
AAL21169.1	201	ltyaigegfsvgg	OmpC

**Table 3:** Natural *Salmonella* class-II epitopes used in this study.

*Salmonella* protein	Sequence
Flagellin1 427–441	VQNRFNSAITNLGNT
Flagellin2 455–469	DSDYATEVSNMSRAQ
SseI 268–280	LIYYTDFSNSSIA
SseJ 329–341	CYYETADAFKVIM

**Figure 2:**
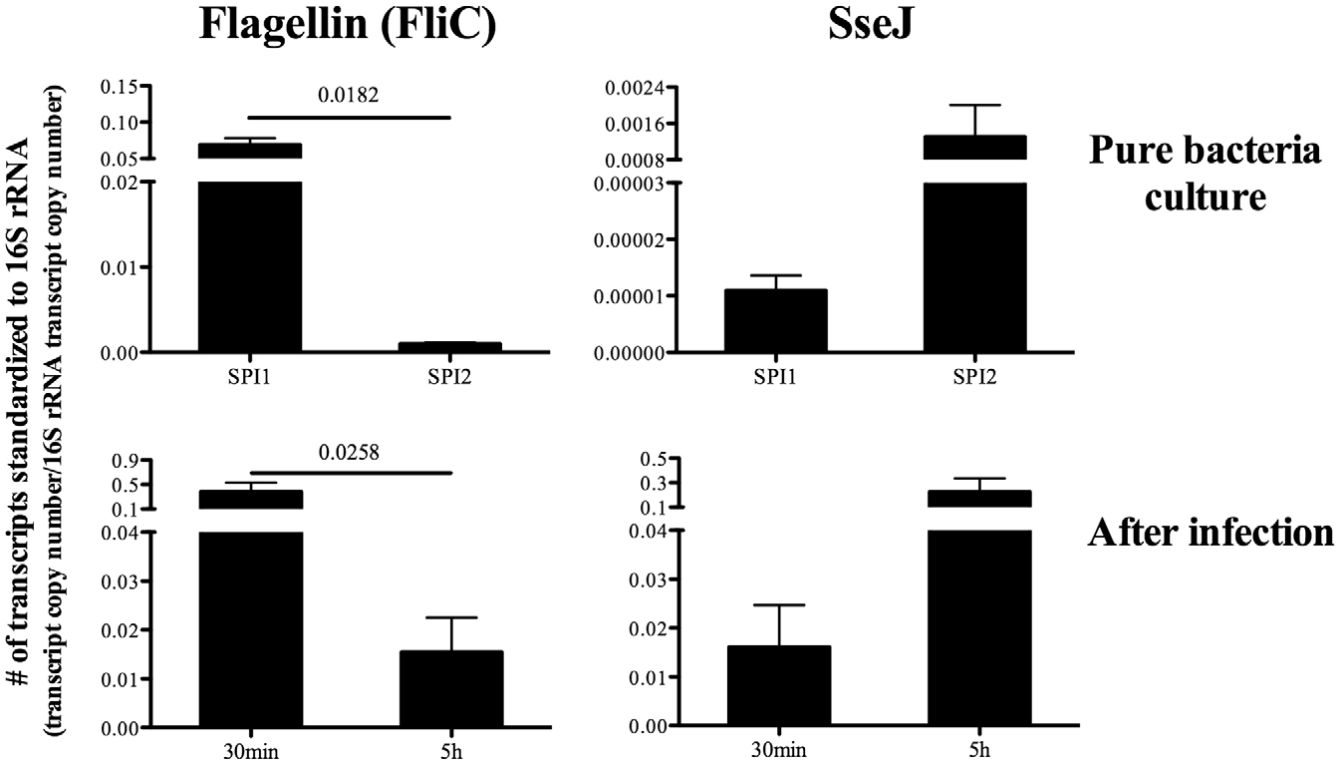
Differential regulation of bacterial Flagellin (FliC) and SseJ in vitro and in vivo. For in vitro detection of bacterial mRNA, *Salmonella* (BRD509) were cultured under SPI1-inducing conditions (LB broth, for 3hours) or SPI2-inducing conditions (modified N minimal medium, for 6hours) (*Top)*. For in vivo detection, C57BL/6 mice were infected intravenously with 5X10^5^ *Salmonella* (BRD509), and spleens harvested 30 min or 5 hours later (*Bottom*). Bacterial RNA was isolated as described in Materials and Methods. Expression of Flagellin (FliC) or SseJ mRNA was quantified by real-time qPCR. Bar graphs show the mean number ± SEM of Flagellin (FliC) or SseJ mRNA transcripts normalized to the respective amount of 16S rRNA in each sample. Data are pooled from two separate experiments (in vitro) or three separate experiments using a total of nine mice (in vivo). Numbers above indicate statistical significance and show the *p* value of a comparison between the groups.

In marked contrast to flagellin, SseI and SseJ are though to be expressed during intra-macrophage replication [35]. We confirmed the differential regulation of flagellin (FliC) and SseJ by examining bacterial mRNA expression in vitro. While FliC mRNA was highly expressed under SPI1-inducing conditions, it was down-regulated under SPI2-inducing conditions which simulate the intra-macrophage environment (Figure 2). In contrast, SseJ was expressed under SPI2-inducing conditions but down-regulated under SPI1-inducing conditions (Figure 2). Thus, S*almonella* differentially regulate the production of flagellin and SseJ, depending on local environmental cues. Although it was technically challenging to measure *Salmonella* mRNA expression in vivo, we were able to confirm the differential regulation of FliC and SseJ at early time points after infection. FliC mRNA was highly expressed at 30 minutes after infection but significantly reduced at 5 hours, while in contrast, SseJ mRNA was low at 5 minutes but expression increased at 30 minutes (Figure 2). Thus, flagellin (FliC) and SseJ represent antigens that are differentially regulated during the transition from extracellular to intracellular growth.

### Construction of *Salmonella*-specific class-II tetramers

**Figure 3:**
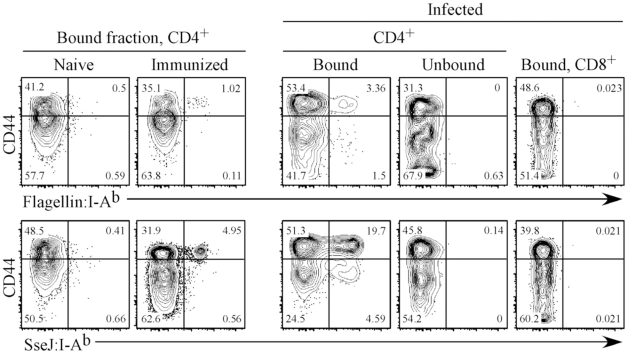
Expansion of endogenous flagellin_427–441_- or SseJ_329–341_-specific CD4 T cells after peptide immunization or *Salmonella* infection of mice. C57BL/6 mice were immunized sub-cutaneously with either 100 µg of flagellin_427–441_ or SseJ_329–341_ peptide in the presence of CFA or infected with 5x10^5^ *Salmonella* (BRD509). On day 7, inguinal, axillary, and brachial lymph nodes were isolated from peptide-immunized mice (Immunized). On day 35, spleens were harvested from C57BL/6 mice infected intravenously with *Salmonella* BRD509 (Infected). Endogenous flagellin_427–441_- or SseJ_329–341_-specific CD4 T cells were detected using Flagellin:I-A^b^ or SseJ:I-A^b^ tetramers, enriched with anti-fluorochrome microbeads, stained with antibodies to several surface markers, and examined by flow cytometry. CD4 T cells from naive, immunized, and *Salmonella*-infected mice, were detected among CD11c^−^CD11b^−^F4/80^−^B220^−^CD3^+^ cells, and further analyzed for expression of CD44 and Flagellin:I-A^b^ or SseJ:I-A^b^ tetramer positive cells. CD4 T cells from the unbound column fraction and CD8 T cells within the bound fraction are also shown as controls. FACS plots are representative of three mice per group and three replicate experiments.

We constructed class-II I-A^b^ tetramers containing flagellin_427–441_ and SseJ_329–341_ epitopes and visualized endogenous flagellin_427–441_-, SseJ_329–341_-specific CD4 T cells using a sensitive tetramer enrichment methodology [36]. C57BL/6 mice contained low numbers of naive flagellin- and SseJ-specific CD4 T cells, but a population of expanded CD44^Hi^ tetramer positive cells was readily detected in the draining lymph nodes after immunization with flagellin_427–441_ or SseJ_329–341_ and CFA (Figure 3). Similarly, infection with *Salmonella* (BRD509) allowed detection of an expanded population of flagellin- and SseJ-specific CD4 T cells (Figure 3). *Salmonella*-specific T cells were not detected in unbound column fractions or in CD8 T cells bound to enrichment columns (Figure 3), demonstrating the specificity of these detection regents and the efficiency of enrichment. We chose to focus on visualizing CD4 T cells responding to *Salmonella* strain BRD509 since this strain has been widely studied previously [37]–[39], and use of more virulent strains precludes analysis at late time points after infection of C57BL/6 mice.

### Distinct kinetics of flagellin- and SseJ-specific CD4 T cell expansion following infection

**Figure 4:**
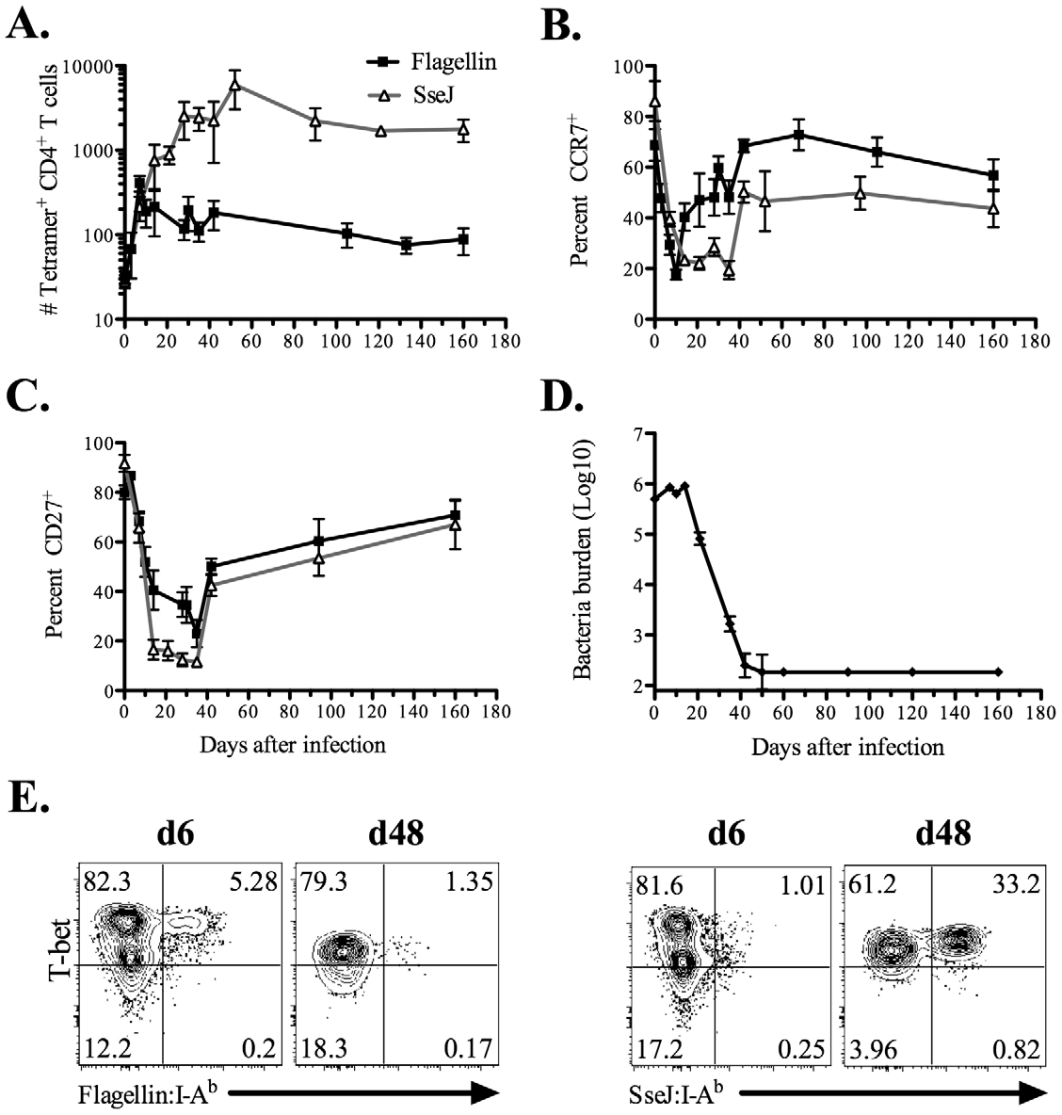
Kinetics of endogenous *Salmonella* flagellin_427–441_-, and SseJ_329–341_-specific CD4 T cells after *Salmonella* infection. C57BL/6 mice were infected intravenously with 5×10^5^ *Salmonella* (BRD509). At various time points, endogenous CD4 T cells were stained using Flagellin:I-A^b^ or SseJ:I-A^b^ tetramers, enriched using magnetic selection, and examined by flow cytometry. CD4 T cells were gated from CD11c^−^CD11b^−^F4/80^−^B220^−^CD3^+^ cells, and further analyzed for Flagellin:I-A^b^ or SseJ:I-A^b^ tetramer positive cells. (A) The number of endogenous flagellin_427–441_- or SseJ_329–341_-specific CD4 T cells was calculated based on FACS analysis. Graph shows the mean number ± SEM of flagellin_427–441_- or SseJ_329–341_-specific CD4 T cells. (B and C) Line graphs show the mean percentage ± SEM of tetramer specific CD4 T cells positive for CCR7 (B) or CD27 (C) expression. (D) The number of viable *Salmonella* determined in the spleen at various time points. These data were pooled from several experiments and represent at least 3–5 mice at each time point. (E) Endogenous flagellin_427–441_- or SseJ_329–341_-specific CD4 were enriched from day 6 or day 48 *Salmonella*-infected mice, and stained intracellularly with antibodies specific for T-bet.

We examined the kinetics of flagellin_427–441_-specific and SseJ_329–341_-specific CD4 T cell expansion after intravenous (IV) infection with *Salmonella*. The pooled secondary lymphoid tissues of a C57BL/6 mouse contained approximately 32 flagellin_427–441_-specific and 30 SseJ_329–341_-specific CD4 T cells (Figure 4A). After *Salmonella* infection, flagellin_427–441_-specific CD4 T cells expanded to a peak of 410 cells at day 7 (12.9 fold expansion over naïve frequency), before contracting to 88 cells by day 160 (Figure 4A). At the peak of clonal expansion, flagellin_427–441_-specific CD4 T cells decreased surface expression of CCR7 and CD27, indicating the development of T effector cells (Figure 4B and C). Flagellin_427–441_-specific T cells started to contract as early as day 10 following infection, and this coincided with a gradual increase in the percentage of cells expressing CCR7 and CD27 (Figure 4A–C). Importantly, this CD4 contraction phase occurred while bacterial burdens remained high in vivo (Figure 4D). In marked contrast, SseJ_329–341_-specific CD4 T cells expanded after infection and continued to accumulate in secondary lymphoid tissues until day 52 post-infection, eventually reaching a peak of 5,900 cells (210-fold expansion over naïve frequency), before decreasing to 1,800 cells by day 160 (Figure 4A). In contrast to the flagellin-specific response, SseJ_329–341_-specific CD4 T cells maintained low expression of CCR7 and CD27 until bacterial clearance had been achieved (Figure 4B–D). We also examined intracellular expression of transcription factors associated with T helper lineage commitment in these two populations at time points close to peak expansion. The vast majority of expanded flagellin_427–441_-specific and SseJ_329–341_-specific CD4 T cells expressed T-bet, while no staining above background levels was detected using antibodies specific for GATA-3, FoxP3, or RORγt (Figure 4E and data not shown).

**Figure 5:**
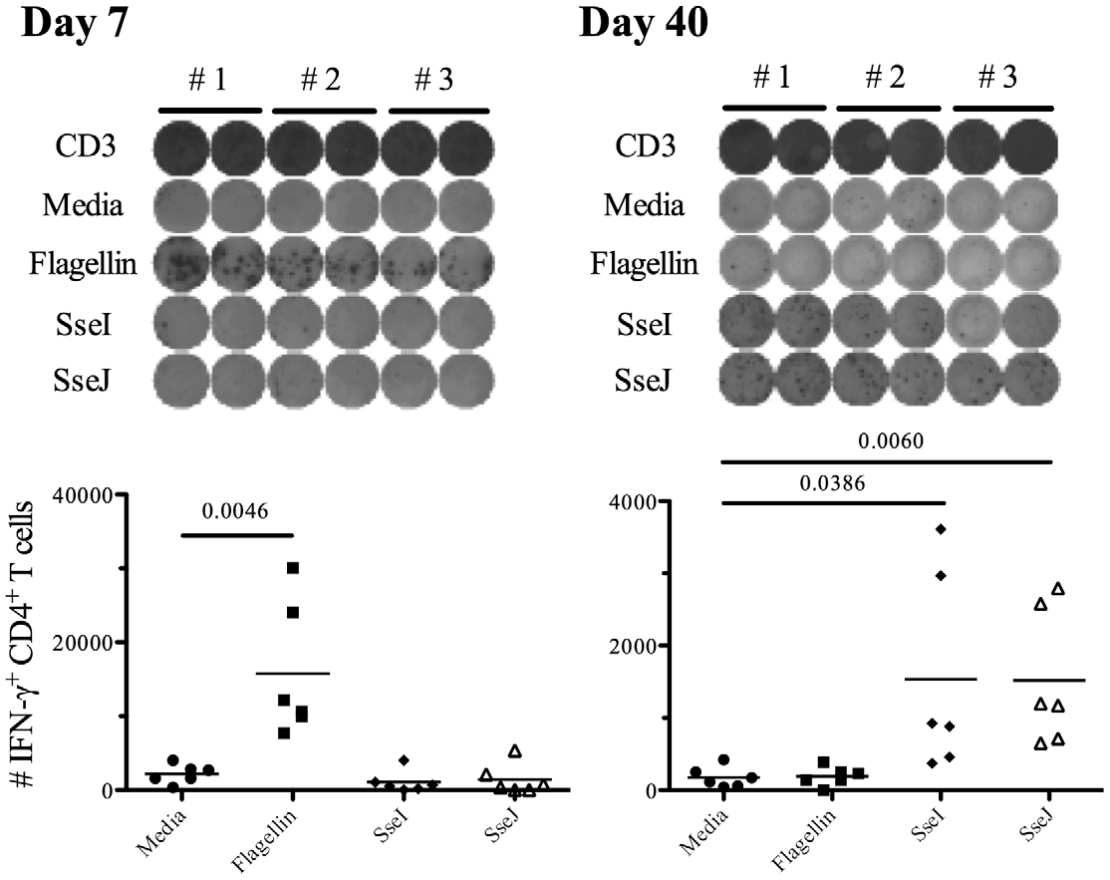
*Salmonella*-specific Th1 cells target flagellin and T3SS effectors at different time points after infection. C57BL/6 mice were infected intravenously with 5×10^5^ Salmonella (BRD509). On day 7 or day 40 post-infection, CD4 T cells from the spleens were purified and restimulated with 10 µM of various peptides (Table 3) for 16 h in the presence of irradiated splenocytes. IFN-γ production was measured by ELISPOT assay. (*Top*) Representative images from IFN-γ ELISPOT plates using CD4 T cells from *Salmonella*-infected mice at day 7 or day 40 after infection. (*Bottom*) Scatter graphs show the number of IFN-γ-producing CD4 T cells per infected mouse. Numbers above indicate statistical significance and show *p* value of a comparison between media alone (no stimulation) and peptide stimulation group.

In order to confirm the distinct tempo of CD4 responses to *Salmonella* flagellin and TTSS effector proteins, we also examined CD4 T cell responses by ELISPOT. IFN-γ-producing CD4 T cells were detected seven days after *Salmonella* infection and were found to predominantly focus on flagellin, rather than TTSS effector epitopes (Figure 5). In marked contrast, 40 days after infection, larger pools of IFN-γ-producing CD4 T cells were detected responding to SseI and SseJ epitopes rather than flagellin (Figure 5). No Th2 or Th17 CD4 T cell response was detected at either of these time points after intravenous infection (data not shown). Together, these data demonstrate distinct temporal differences in the targeting of flagellin and TTSS effector proteins by *Salmonella*-specific CD4 T cells.

### Bacterial persistence is required for accumulation of SseJ-specific T cells

**Figure 6:**
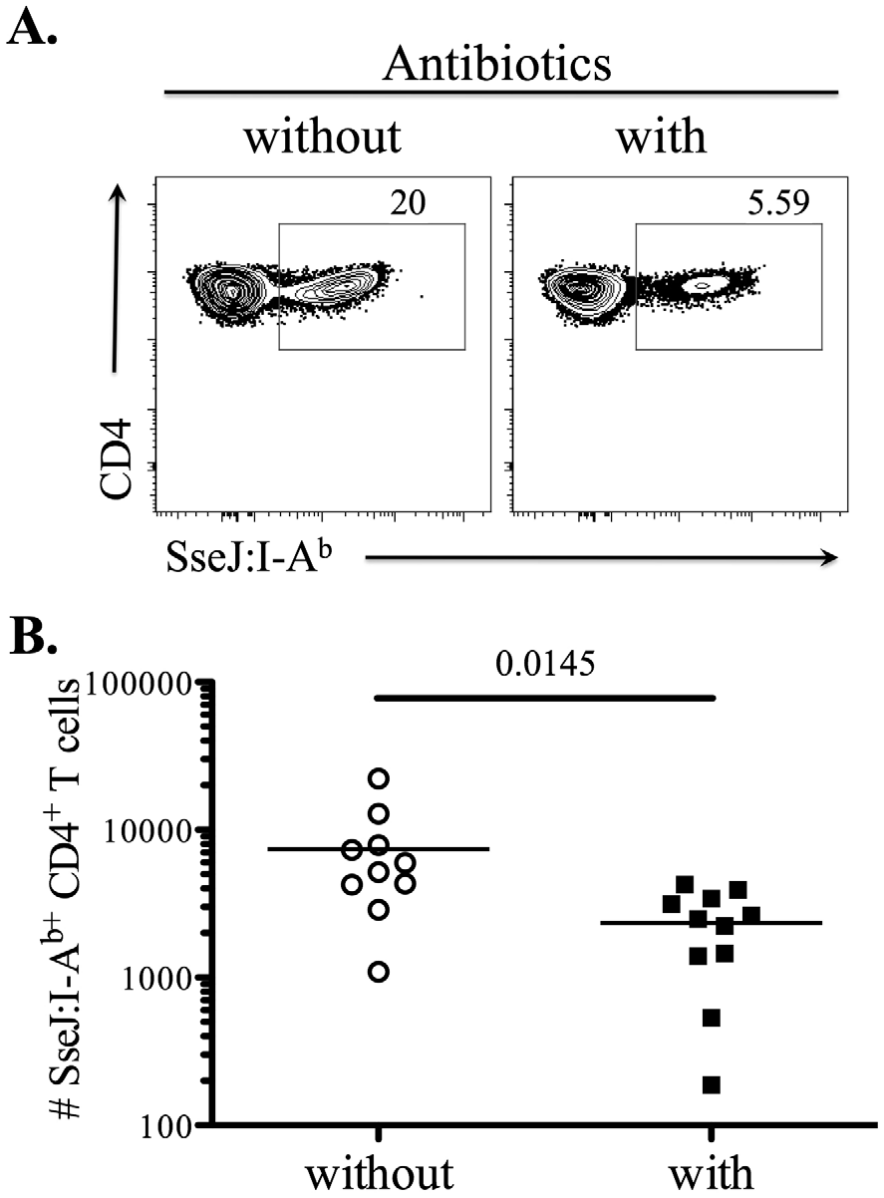
Bacterial persistence is required for optimal expansion of SseJ-specific CD4 T cells. C57BL/6 mice were infected intravenously with 5×10^5^ *Salmonella* (BRD509). Some mice were treated with Enrofloxacin (Baytril) (2mg/ml in drinking water), beginning 5 days post-infection. At day 30 post-infection, endogenous SseJ-specific CD4 T cells were stained with SseJ:I-A^b^ tetramers, enriched, and examined by flow cytometry. (A) Representative FACS plots from control (without, *left*) or antibiotic-treated mice (with, *right*). Tetramer-specific CD4 T cells were gated from CD11c^−^CD11b^−^F4/80^−^B220^−^CD3^+^. (B) Scatter plots show the number of SseJ_329–341_-specific CD4 T cells in control (without) or treatment group (with). Data are pooled from three separate experiments. Numbers above indicate statistical significance and show *p* value of a comparison between control and treatment group.

We hypothesized that the temporal difference in the CD4 T cell response to flagellin and SseJ was due to the maintenance of SseJ expression in vivo by persistent bacteria. However, it was also possible that these different kinetics reflected T cell-intrinsic variables that were completely unrelated to antigen persistence. We therefore examined the clonal expansion of SseJ-specific T cells in mice that were administered antibiotics to clear bacteria, beginning five days after infection. Although SseJ-specific T cells were clearly detected in both treated and untreated mice, clonal expansion was significantly reduced in mice that had been treated with antibiotics (Figure 6). Thus, the continued accumulation of SseJ-specific CD4 T cells at later time points after infection required bacterial persistence.

### Enrichment of flagellin-specific Th17 CD4 T cells in mucosal tissues after oral infection

**Figure 7:**
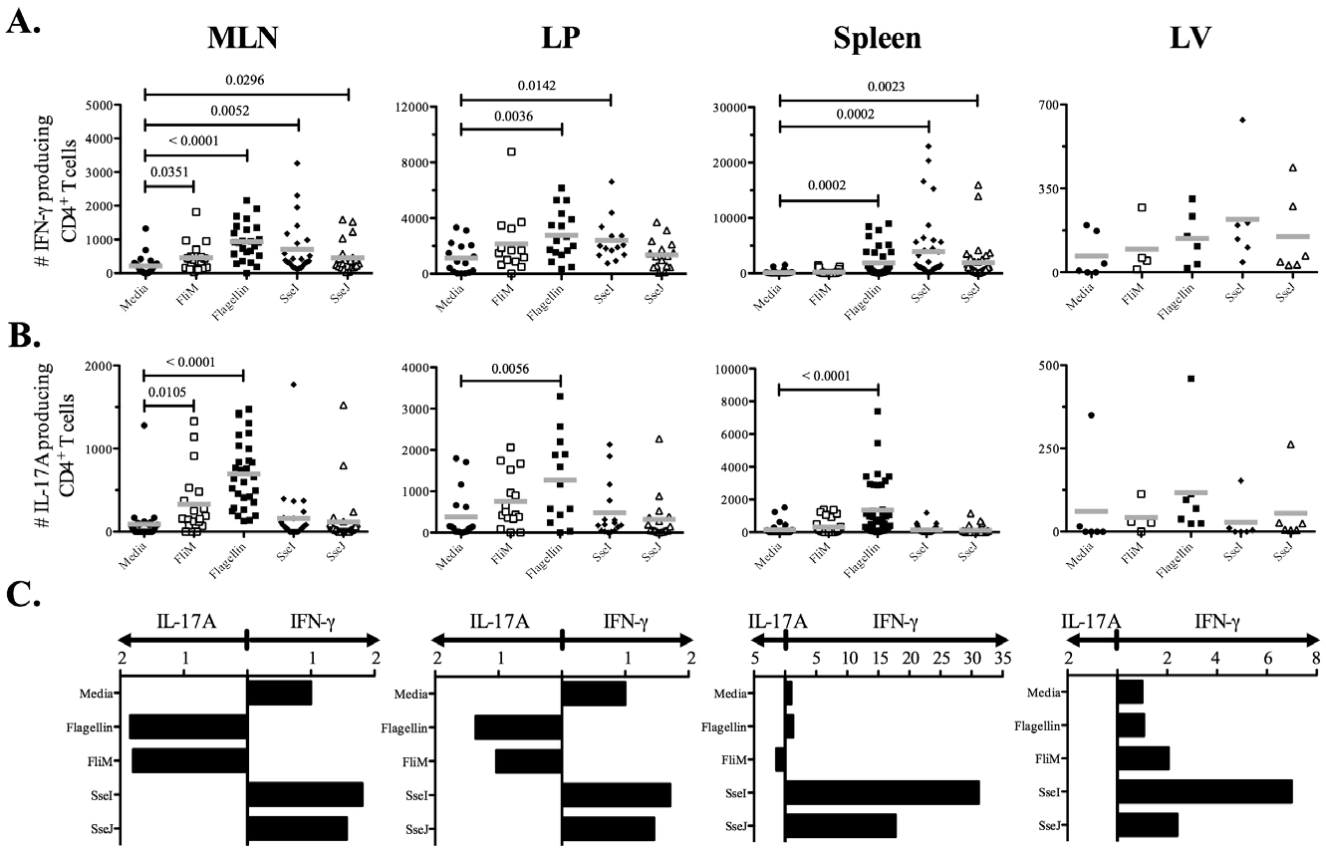
Detection of flagellin-specific CD4 Th17 cells in intestinal tissues. C57BL/6 mice were infected three times orally with 5×10^9^ *Salmonella* (BRD509) at one-month intervals. On day 7 after the third infection, CD4 T cells from the spleens, MLN, including Peyer's patches, livers (LV), and laminar propria (LP) were purified. Isolated CD4 T cells (1×10^5^) were restimulated with 10 µM of various peptides (Table 3) for 16 h in the presence of irradiated splenocytes. (A) IFN-γ or, (B) IL-17A production was measured by ELISPOT. Scatter graphs show the number of (A) IFN-γ or, (B) IL-17A-producing CD4 T cells in infected tissues from individual mice. (C) Bar graphs show the mean ratio of IFN-γ to IL-17 production after in vitro stimulation. Numbers above indicate statistical significance and show *p* value of a comparison between media alone (no stimulation) and peptide stimulation group.

**Figure 8:**
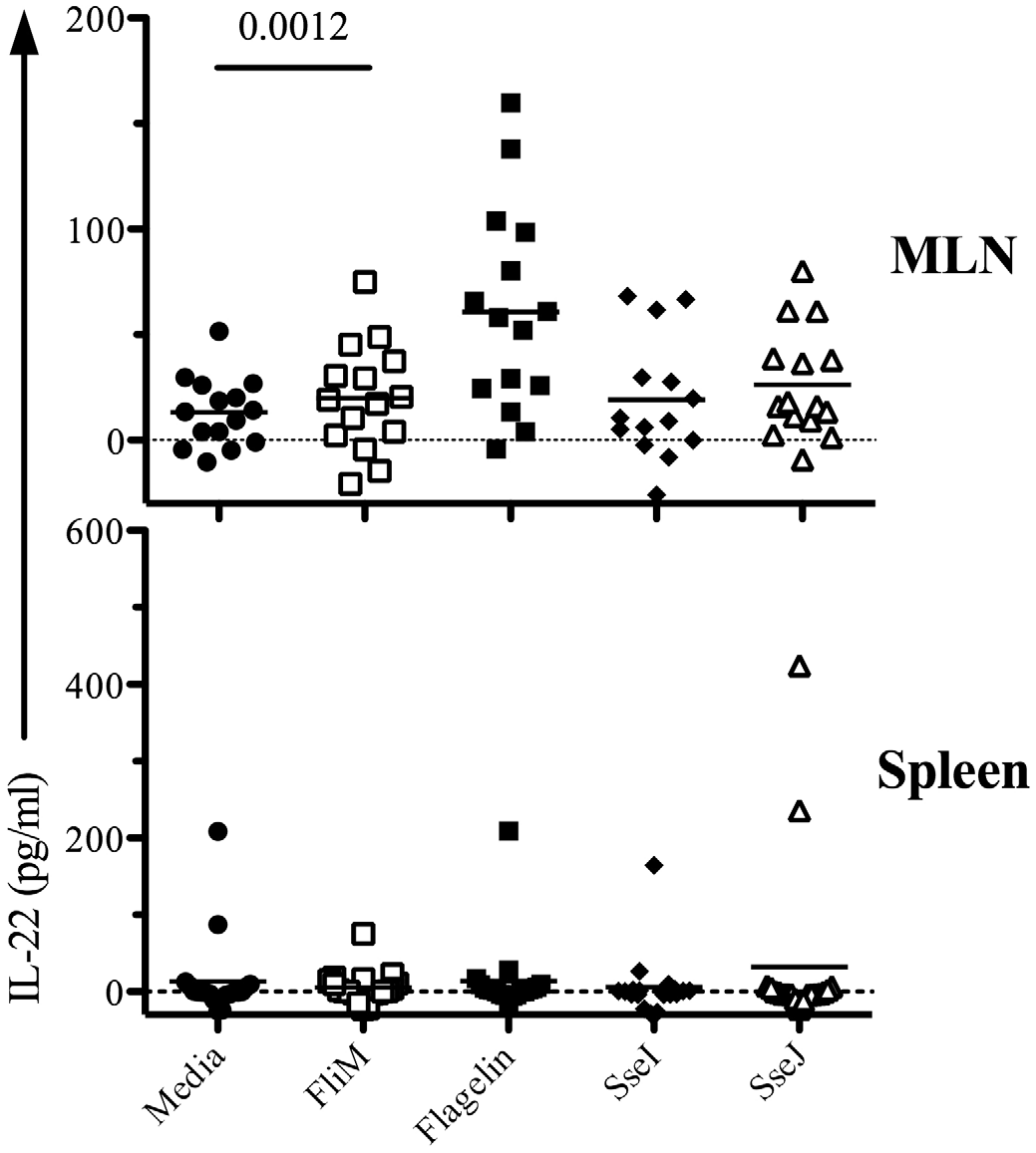
Mucosal flagellin-specific CD4 T cells produce IL-22 upon restimulation. C57BL/6 mice were infected three times orally with 5×10^9^ Salmonella (BRD509) at one-month intervals. On day 7 after the third infection, CD4 T cells from spleen and MLN, including Peyer's patches, were isolated. Purified CD4 T cells were restimulated with 10 µM peptide (Table 3) for 16 h in the presence of irradiated splenocytes. Culture supernatants were collected and IL-22 production measured by ELISA. Data show IL-22 production in culture supernatants as scatter plots representing individual mice. Data are pooled from three separate experiments. Numbers above each group indicate statistical significance and show the *p* value of a comparison between media alone (no stimulation) and peptide stimulation group.

Next, we used ELISPOTs to examine the CD4 T cell response to oral *Salmonella* infection and closely monitored Th1, Th2, and Th17 responses to flagellin and TTSS effector proteins in mucosal and systemic tissues. We used three oral doses of *Salmonella* (BRD509) as this was required for optimal priming of effector CD4 T cell responses. Consistent with the absence of Th2 responses after IV infection, IL-4 production was not detected in either mucosal or systemic tissues (data not shown). In contrast, IFN-γ-producing CD4 T cells were detected in intestinal (mesenteric lymph nodes, MLNs, and lamina propria, LP) and systemic (spleen and liver) tissues (Figure 7A). SseI and J-specific CD4 Th1 cells were more numerous in the spleen and liver, while flagellin-specific CD4 Th1 cells were predominantly detected in intestinal tissues (Figure 7A). Furthermore, IL-17A-producing CD4 T cells were also detected and this response was notably focused on the flagellin epitopes and occurred in mucosal tissues (Figure 7B). Indeed, flagellin-specific CD4 T cells in intestinal tissues had a higher ratio of Th17:Th1 cells, while in contrast SseI and J-specific CD4 T cells in systemic tissues had a higher ratio of Th1:Th17 cells (Figure 7C). In order to confirm the preferential generation of flagellin-specific Th17 cells in the intestine, we also examined IL-22 production by CD4 T cells recovered from the spleen or MLN of *Salmonella*-infected mice. Consistent with the ELISPOT data, IL-22 was detected after stimulation of CD4 T cells from the MLN but not the spleen of infected mice, and was directed towards flagellin_427–441_ rather than T3SS effectors (Figure 8).

## Discussion

This study describes the first examination of endogenous CD4 T cell responses to multiple *Salmonella* epitopes using tetramer and ELISPOT assays. Previous analysis of *Salmonella*-specific CD4 T cells has been severely limited by the lack of defined epitopes and has largely focused on the response to a single I-A^b^ epitope within flagellin [13], [40], [41]. Our ability to track multiple epitope-specific CD4 responses in this study has revealed that in vivo regulation of bacterial antigen expression is a critical factor in orchestrating both the tempo and effector maturation of *Salmonella*-specific CD4 T cells at mucosal and systemic sites. High expression of flagellin is reported from extracellular bacteria but this is rapidly decreased after infection of macrophages, and flagelin cannot be detected in the spleen of infected mice [32], [42]. In contrast, expression of SPI-2 genes is initiated within the phagolysosome of infected macrophages and is required for bacterial survival in vivo [35]. Our data confirm these findings and demonstrate differential expression of FliC and SseJ mRNA in response to environmental cues, both in vitro and in vivo. Thus, naïve *Salmonella*-specific CD4 T cells will encounter flagellin only during the early stage of infection within the intestine, whereas SPI-2 TTSS effector proteins accumulate as more intracellular bacteria replicate in the systemic tissues of the spleen and liver.

Interestingly, our tetramer data reveal a pattern of CD4 T cell expansion and contraction to flagellin and SseJ that corresponds closely to their relative temporal abundance during in vivo growth. In particular, we detected early contraction of flagellin-specific T cells, while SseJ-specific T cell responses remained elevated for several weeks. There may be T cell-intrinsic factors that determine the peak clonal expansion of FliC- and SseJ-specific T cells since SseJ-specific T cells exhibit greater clonal expansion. However, this issue does not explain the maintenance of the SseJ response for several weeks after infection. Furthermore, the elimination of bacteria using antibiotics severely dampened the CD4 T cell response to SseJ, indicating that antigen persistence is essential for sustained clonal expansion of this population. Although antibiotics can affect adaptive response indirectly via effects on commensal flora, it is more likely that the reduction in the SseJ response is due to a direct effect on bacterial persistence. Together, these data suggest that CD4 T cells are surprisingly dependent upon local antigen availability even after clonal expansion has occurred and indicate that clearer definition of in vivo protein expression by pathogens might be critical for identification of protective antigens, especially for pathogens that reside in multiple tissues or have distinct life-cycle stages. Indeed, a correlation between temporal antigen expression and in vivo activation of protective CD4 T cell responses has recently been observed during *M. tuberculosis* infection [43], [44]. Furthermore, a requirement for sustained antigen expression for the generation of optimal CD4 T cell responses has also been identified in other model systems [45], [46].

Our data also demonstrate divergent CD4 T cell helper development to flagellin and TTSS effector proteins. After intravenous infection, CD4 T cells responding to each of these antigens expressed T-bet and produced IFN-γ following in vitro restimulation, thus *Salmonella* generates distinct antigen-specific Th1 cell populations that appear at different stages of infection. This finding may help explain why Th1 cells can be detected within days of *Salmonella* infection but do not appear to actively participate in bacterial clearance until several week later [47]. If the majority of the early *Salmonella*-specific Th1 response is largely focused on antigens that are transiently expressed, such as flagellin or SPI-1 gene products, then intracellular bacteria can evade detection by the initial wave of Th1 effector cells. In contrast, a second wave of Th1 cells that are specific for highly expressed intracellular antigens such as T3SS effectors would be more likely to participate in the later stages of bacterial clearance from systemic tissues. A similar model of bacterial antigen regulation and effector T cell evasion has been proposed in mycobacterial infection [43], [48], and may be a common feature of persistent bacterial infection.

After oral infection with *Salmonella*, we detected Th17 cells specific for flagellin epitopes and these were particularly enriched in the intestine. TGF-β is abundant in mucosal tissues and plays an important role in the generation of both Treg and Th17 cells [5]. Intestinal IL-6 production is induced by *Salmonella* infection and is likely to drive *Salmonella*-specific differentiation towards a Th17 lineage in the intestine [49], [50]. Indeed, flagellin is a ligand for TLR5 and can directly induce IL-6 production in vitro and in vivo [38], [51]. This may explain why Th17 cells specific for flagellin epitopes found only in infected intestinal tissues while Th17 responses to T3SS effector proteins are largely absent. Th17 cells are thought to provide protective immunity during extracellular bacterial infections via upregulation of anti-bacterial mediators and neutrophil recruitment [5], [52]. Since *Salmonella* grow intracellularly in vivo [53], a protective role for *Salmonella*-specific Th17 CD4 T cells during primary infection is not immediately obvious. However, Th17 cells may be vital for mobilizing local innate defenses against secondary infection after bacteria penetrate intestinal epithelium. Indeed, it may be particularly important that these mucosal Th17 cells recognize bacterial antigens such as flagellin that are expressed by the initial bacterial burden.

In summary, this study has identified new *Salmonella* I-A^b^ epitopes in T3SS effectors and used peptide-MHC tetramers and ELIPOT assays to simultaneously track multiple CD4 T cell responses during *Salmonella* infection for the first time. These data show that an infected host develops distinct CD4 effector lineages that are tightly regulated by bacterial antigen expression patterns in vivo, a finding that may assist the generation of vaccines directed against microbial pathogens that replicate in mucosal and systemic tissues.

## Materials and Methods

### Ethics statement

This study was carried out in strict accordance with the recommendations in the Guide for the Care and Use of Laboratory Animals of the National Institutes of Health. The University of Minnesota and University of California Davis are accredited by the Association for Assessment and Accreditation of Laboratory Animal Care (AAALAC). All animal experiments were approved by University of Minnesota Institutional Animal Care and Use Committee (IACUC) (Protocol numbers: 1011A93173 and 1004A80157) or UC Davis IACUC Protocol 16612.

### Mice and bacterial strains

C57BL/6 and 129Sv mice were purchased from the National Cancer Institute (Frederick, MD) and used at 6–16 weeks of age. All mice were maintained in accordance with University of Minnesota or University of California Davis Research Animal Resource guidelines. *Salmonella enterica* serovar Typhimurium strains SL1344 and BRD509 (AroA^−^D^−^) were kindly provided by Dr. D. Xu (University of Glasgow, Glasgow, U.K).

### Salmonella infection and antibiotic treatment

*S.* Typhimurium were cultured overnight in Luria-Bertani (LB) broth without shaking, and diluted in PBS after estimation of bacterial concentration using a spectrophotometer. Mice were infected intravenously in the lateral tail vein with 5X10^5^ BRD509. For oral infection, mice were administered 0.1ml of 5% sodium bicarbonate to neutralize stomach pH before oral infection with 5x10^9^ BRD509. The actual bacterial dose administered was confirmed by plating serial dilutions of the original culture onto MacConkey agar plates counting the number of colonies that grew after overnight culture at 37°C. In some experiments mice were administered Enrofloxacin (Baytril) at 2mg/ml in their drinking water, beginning on 5 days post-infection, as previously described [54].

### *Salmonella* I-A^b^ epitope discovery

The crystal structure of the murine I-A^b^ molecule in complex with human class II invariant chain-associated peptide (CLIP) [33], was used to identify potential I-A^b^ epitopes from *S.* Typhimurium. Based on the proposed alignment, the likelihood of a particular amino acid residue appearing in a specific position in the MHC class-II binding groove was assigned a numerical score. We focused on binding positions 1, 4, 6, 7, and 9 as these were the most likely to contribute to MHC class-II binding. For example, a tyrosine at position 1 was found in 30 out of 75 core residues examined and was assigned a score of 30. An overall score total was calculated by adding individual scores from each potential binding residue, with 109 being the highest possible score. This scoring approach was applied bioinformatically to each open reading frame of *Salmonella* using 9-mers overlapping by one (a total of 995,592 peptides). Two algorithms were then applied to this data; the first was a position-specific scoring matrix (PSSM) [55], and the second a hidden Markov model (HMM) [56]. The epitopes with highest PSSM (25 epitopes from 105 to 84) and HMM scores (19 epitopes from 82 to 43) were tested for immunogenicity using an IFN-γ ELISPOT assay. Briefly, four groups of eleven peptides were mixed in equal amounts (5 mg/peptide) emulsified in complete Freund's adjuvant (CFA) and injected subcutaneously into groups of C57BL/6 mice. Draining lymph nodes were harvested eight days later, CD4 T cells enriched by negative selection, and mixed 1∶1 with irradiated splenocytes in IFN-γ ELISPOT plates. Duplicate wells were stimulated for 36 hours with individual peptides and spot forming cells (SFC) per 1x10^6^ total cells enumerated. Although 88% (PSSM) and 74% (HMM) of peptides induced a CD4 T cell response in HKST/CFA-immunized mice, no response to these epitopes was detected after infection with *Salmonella*. The approach was therefore modified using PSSM to identify the two highest scoring candidate peptides from a combination of 13 outer membrane and secreted proteins. These epitopes were tested using purified CD4 T cells from the spleen and mesenteric lymph nodes of 129Sv mice orally infected 11 weeks previously with 5x10^7^ *Salmonella* (SL1344). In these peptide identification experiments, genetically resistant 129Sv mice were used so that CD4 responses to virulent (SL1344) bacteria could be examined at late time points when C57BL/6 mice would normally succumb to infection.

### Quantifying Flagellin (FliC) and SseJ mRNA

For SPI1 or SPI2-inducing cultures, *Salmonella* BRD509 were grown overnight at 37°C in LB broth, diluted in either LB broth for SPI1-inducing conditions or modified N minimal medium [57] for SPI2-inducing conditions, and grown for either 3 hours for SPI1 or 6 hours for SPI2 conditions. For in vivo detection of mRNA expression, C57BL/6 mice were infected intravenously with 5X10^5^ BRD509 and spleens were harvested at either 30 minutes or 5 hours after infection. Total RNA was isolated using Aurum Total RNA Mini Kit (Bio-Rad, Hercules, CA) or TRI reagent (Applied Biosystems/Ambion, Austin, TX) according to the manufacturer's protocol. To enrich bacterial RNA from infected splenocytes, the MICROBEnrich kit (Ambion) was used, and traces of genomic DNA eliminated using DNA-free DNase treatment kit (Ambion) according to manufacturer's recommendations. To quantitate expression of *Salmonella* Flagellin (FliC) or SseJ by real-time qPCR, 1 µg of bacterial RNA was used as a template and cDNA synthesis by random hexamers was performed using TaqMan reverse transcription reagents (Applied Biosystems). SYBR Green (Applied Biosystems) based real-time qPCR was carried out based on published procedures [58]. The following primers were used in this study; 16S rRNA, 5′-tgttgtggttaataaccgca-3′ and 5′-gactaccagggtatctaatcc-3′ [59]; FliC, 5′-gtaacgctaacgacggtatc-3′ and 5′-atttcagcctggatggagtc-3′ [58]; SseJ, 5′-tattacgagactgccgatgc-3′ and 5′-gcccgtggtgagtataagggt-3′ (GeneScript's real-time PCR primer design tool). Data was acquired using a ViiA 7 Real-time PCR system (Applied Biosystems) and analyzed using comparative Ct method (Applied Biosystems). Transcripts of Flagellin (FliC) or SseJ were normalized to the respective amounts of 16S rRNA in each sample.

### Tracking Salmonella specific endogenous CD4 T cell response using class-II tetramers

Endogenous *Salmonella* specific flagellin_427–441_ - or SseJ_329–341_ -CD4 T cell responses were monitored using a previously described methodology. Biotinylated I-A^b^ monomers with an attached peptide derived from *Salmonella* flagellin_427–441_ (Flagellin:I-A^b^) or SseJ_329–341_ (SseJ:I-A^b^) were produced in S2 Drosophila insect cells cultured using a Wave Bioreactor (GE Healthcare Biosciences, Pittsburgh, PA). Purified monomers were tetramerized using fluorochrome-conjugated streptavidin and batch-tested for optimal binding to CD4 T cells from day 7 peptide-immunized mice, as previously described [36]. Briefly, C57BL/6 mice were immunized with either 100 µg of flagellin_427–441_ or SseJ_329–341_ peptide in the presence of CFA (Sigma-Aldrich, St. Louis, MO). On day 7, the inguinal, axillary, and brachial lymph nodes were harvested from naïve or peptide-immunized mice and stained with respective class-II tetramers in the presence of Fc block (culture supernatant from the 24G2 hybridoma, 2% mouse serum, 2% rat serum, and 0.01% sodium azide). Cells were incubated with anti-fluorochrome microbeads (Stem cell technologies, Vancouver, Canada) and tetramer-specific cells enriched using a magnet. Bound and unbound fractions were stained with fluorochrome-conjugated Abs specific for CD3, CD4, CD8, CD11b, CD11c, B220, F4/80, CCR7, and CD27 (eBioscience, or BD Bioscience, San Diego, CA). For transcription factor staining, cells were surface stained, and then treated with Foxp3 staining buffer set (eBioscience). Permeabilized cells were stained with fluorochrome-conjugated Abs specific for T-bet, GATA3, RORγT, or Foxp3. Cells were then analyzed by flow cytometry using an LSR II (BD Bioscience) or FACS Canto (BD Bioscience). Endogenous tetramer specific CD4 T cells were identified using a previously described gating strategy [36]. Tetramer specificity was confirmed by the absence of tetramer binding CD8 T cells in the bound fraction and in CD4 T cells within the unbound fraction. All the data were analyzed using FlowJo software (Tree Star, San Carlos, CA).

### Cell processing for ELISPOT assay

Spleen and MLN, including Peyer's patches, were crushed through nylon mesh, and then RBC in spleen was lysed with ACK lysis buffer (Lonza, Walkersville, MD). For nonlymphoid tissue processing, we followed published methods [60]. Livers were perfused, crushed through a cell strainer (BD Biosciences), and resuspended in 35% Percoll (Sigma-Aldrich). After centrifugation, pelleted cells were treated with ACK lysis buffer and then washed with 2% FBS/PBS. For Lamina propria (LP) lymphocytes, small intestine was dissected, and washed with 2% FBS/CMF (Ca^2+^ and Mg^2+^ free HBSS with 1mM HEPES, and 2.5mM NaHCO_3_). The intestine pieces were then stirred at 37°C for 30 min in CMF containing 10% FBS and 1mM dithioerythritol. The epithelial cells were removed by stirring the pieces in 1.3 mM EDTA/HBSS solution at 37°C for 30 min. The intestinal tissues were then treated with collagenase (Invitrogen, Carlsbad, CA) in RPMI 1640 (with 1mM CaCl_2_, 1mM MgCl_2_, and 5% FBS) at 37°C for 1 h. Cells were washed in 2% FBS/PBS, and then centrifugated on a 44%/67% Percoll gradient (GE Healthcare Biosciences). Viable cells at the interface were collected, and these cells are LP lymphocytes.

### Examining *Salmonella* specific endogenous CD4 T cell response using ELISPOT assay

Multiscreen_HTS_ ELISPOT plate (Millipore, Billerica, MA) was coated with either purified anti-mouse IFN-γ (BD Bioscience) or IL-17A (eBioscience). Total CD4 T cells from each tissue were isolated using EasySep Mouse CD4 T cell enrichment kit (Stem cell technologies) according to the manufacturer's protocol. The purity of CD4 T cells were measured by flow cytometry and was typically >90% for splenocytes and MLN, and >70% for non-lymphoid tissues. Purified CD4 T cells (10^5^ cells/well) were added to ELISPOT plates in the presence of 4×10^5^ cells irradiated splenocytes. Cells were restimulated with 10 µM peptides (Table 3) at 37°C overnight. Bound IFN-γ or IL-17A was detected by biotin-conjugated anti-mouse IFN-γ (BD Bioscience) or IL-17A (eBioscience), followed by AP-conjugated streptavidin (BD Bioscience). Bound antibodies were visualized using One-step NBT-BCIP substrate (Thermo, Rockford, IL), and plates analyzed using an ELISPOT reader ImmunoSpot (Cellular Technology, Shaker Heights, OH).

### IL-22 ELISA

Purified CD4 T cells (1×10^5^ cells/well) from spleens and MLN, including Peyer's patches, were restimulated with 10 µM peptides (Table 3) in the presence of 4×10^5^ irradiated splenocytes for 16 h. IL-22 secretion was measured from culture supernatant using mouse IL-22 ELISA Ready-SET-Go kit (eBioscience). Briefly, High-protein binding plates (Costar, Corning, NY) were coated overnight with capture IL-22 antibody. After incubation in Assay Diluent for 1 hour at room temperature, plates were washed twice before culture supernatant was added. After incubation for 2 hours, plates were washed and biotin-conjugated detection IL-22 antibody added. After 1 hour, plates were washed and incubated for 30 min with avidin-HRP. Bound antibody was visualized using TMB substrate and after 15 min, the reaction stopped by adding 50 µl of 2N H_2_SO_4_. Plates were analyzed using a spectrophotometer (SpectraMax M2, Molecular Devices, Sunnyvale, CA).

### Statistical analysis

Statistical differences between groups of normally distributed data were examined using Prism (GraphPad Software, La Jolla, CA). Data in each group were compared using an unpaired *t* test and were considered significantly different with a *p<0.05*.
